# Green approach for the recovery of secondary metabolites from the roots of *Nardostachys Jatamansi* (D. Don) DC using microwave radiations: Process optimization and anti-alzheimer evaluation

**DOI:** 10.3389/fpls.2022.987986

**Published:** 2022-11-01

**Authors:** Ashwani Arya, Rubal Chahal, Mikhlid H. Almutairi, Deepak Kaushik, Lotfi Aleya, Mohamed Kamel, Mohamed M. Abdel-Daim, Vineet Mittal

**Affiliations:** ^1^ Department of Pharmaceutical Sciences, Maharshi Dayanand University, Rohtak, Haryana, India; ^2^ Department of Zoology, College of Science, King Saud University, Riyadh, Saudi Arabia; ^3^ Chrono-Environnement Laboratory, UMR CNRS 6249, Bourgogne, Franche-Comté University, CEDEX, Besançon, France; ^4^ Department of Medicine and Infectious Diseases, Faculty of Veterinary Medicine, Cairo University, Giza, Egypt; ^5^ Pharmacology Department, Faculty of Veterinary Medicine, Suez Canal University, Ismailia, Egypt

**Keywords:** *Nardostachys jatamansi*, antioxidant, scanning electron microscopy, microwave assisted extraction, gas chromatography, response surface methodology

## Abstract

*Nardostachys jatamansi* (D. Don) DC is a highly valued medicinal herb that has been used in traditional medicinal systems for its remedial effects. Owing to the over-exploitation and unethical trade of *N. jatamansi*, the accelerating global demand of herbal products from this plant cannot be satisfied by the conventional extraction approach. In view of the progressive demand and incredible biological potential of herb, the present research was designed to optimize various extraction parameters for microwave-assisted extraction (MAE). The extracts obtained from the traditional and green approach were also assessed for the recovery of secondary metabolites and anti-Alzheimer’s potential. Various parameters like microwave power, temperature, and time of irradiation were optimized for MAE using Box Behkhen Design (BBD) The scanning electron microscopy of different plant samples was also done to observe the effect of microwave radiations. Further, the metabolite profiling of different extracts was also done by gas chromatography-mass spectrometry (GC-MS) analysis. Also the different behavioral and biochemical parameters along with acetylcholinesterase (AChE) inhibitory potential were assessed to evaluate the anti-Alzheimer’s potential. Optimized parameters for MAE were found to be as microwave power 187.04 W, temperature 90°C, and irradiation time 20 min. The extract yield in MAE was significantly enhanced as compared to the conventional method. Also, the total phenolic content and total flavonoid content (TFC) were improved pointedly from 32.13 ± 0.55 to 72.83 ± 1.1 mg of GAE/g of extract and 21.7 ± 0.85 to 39.21 ± 0.7 mg of RUE/g of extract respectively. Later, the GC-MS analysis of various extracts confirmed the enhancement in the concentration of various sesquiterpenes like jatamansone, spirojatamol, valerenal, valeric acid, globulol, nootkatone and steroidal compounds such as sitosterol, ergosterol, stigmastanone, etc. in the optimized extract. A significant improvement in anti-Alzheimer’s potential was also observed owing to the better concentration of secondary metabolites in the optimized microwave extract. From the current findings, it could be concluded that the MAE could be a successful and green alternative for the extraction and recovery of secondary metabolites from the selected medicinal herb.

## 1 Introduction

A vast range of secondary metabolites is produced by plants which may act as the compounds of defense against herbivores, microbes, and other plants or may act as the signal molecules, UV protectants, and antioxidants (Pagare et al., 2015; Wink, 2015; Badyal et al., 2020). Secondary metabolites from plants, by and large, owe an impressive range of biological and therapeutic possessions. Consequently, plants and their extracts have thus been and are still utilized as remedies to treat various infections, health disorders, and ailments or as flavors, perfumes, dyes, dietary supplements, toxins, and pesticides (Wink, 2015; Teoh, 2016; Alamgir, 2018; Velu et al., 2018). Importantly, the plants are renewable assets for providing raw materials and phytochemicals (precisely, secondary metabolites) for various industrial and pharmaceutical applications. For these reasons, plants are supposed as vital to favor the evolution to a low-cost bio-economy that is not fossil-resource dependent. Moreover, currently, the global market for herbal medicines is steadily growing and is estimated to achieve 7.2% CAGR for the next five years. Such considerable economic, as well as remedial potential of secondary metabolites from plants or their extracts, has triggered the hunt for novel or improved ways to extract the plants. Moreover, the exploration of innovative methods for the extraction of plants for secondary metabolites is needed to meet the growing demand of the herbal industry (Guerriero et al., 2018).

Further, it has been observed that most of the herbal products coming from India are prepared by using Himalayan plants. As the Himalayan flora found to possess an immense collection of phytochemicals with well-known pharmacological potential, and are exceptionally appropriate for all herbal sectors including healthcare, foods, beverages, nutraceuticals, and personal care (Dhiman and Bhattacharya, 2020). The selected medicinal plant, *Nardostachys jatamansi* (D. Don) DC (Family- Capriofoliaceae) is also one of the commercially important herbs from the Himalayan region. The plant is mainly collected from wild sources as the cultivation of this herb is not so successful. However, the altitude of 2200-4800 m and sandy loamy soil with sufficient moisture is favorable for its growth (Nautiyal et al., 2003; Ghimire and Aumeeruddy-Thomas, 2009). This species bears an extended history of use and has been extensively mentioned in Unani, Ayurveda, Nepalese, Bhutanese, Japanese, and Chinese systems of medicine. The herb was also used as a seasoning in a sweet and spiced wine drink called “Hippocras” (Bose et al., 2019; Kaur et al., 2020). In traditional medicine systems, this species is considered a mind rejuvenator or brain tonic, stimulant, antidiabetic, cardio tonic, antipyretic, and antiflatulent, and is used in treating various neurological disorders like epilepsy, insomnia, anxiety, depression, and hysteria. It also claimed to exhibit antioxidant, antimicrobial, hepatic- and cardio-protective, and anticancer properties (Rao et al., 2005; Ahmad et al., 2006; Subashini et al., 2007; Sharma and Singh, 2012; Sahu et al., 2016; Kapoor et al., 2017; Saroya and Singh, 2018). The pharmacological potential of the plant is attributed to the presence of various secondary metabolites of different classes like sesquiterpenes, phenolics, flavonoids, steroids, lignans, coumarins, etc (Bose et al., 2019; Singh, 2020). Moreover, a large number of herbal pharmaceutical companies are commercially marketing single or polyherbal preparations from this plant globally (Dandagi et al., 2008; Thorat et al., 2009). Also, Ved and Goraya (2007) estimated the annual trade for its rhizomes and extract in domestic markets has increased to 200-500 metric tons. But the great demand for the plant actives and extract from this herb cannot be fulfilled by the conventional techniques alone. Traditional methods such as maceration, percolation, and distillation were generally applied for the extraction of herbs. However, these processes are not environmentally friendly and require a lot of time and solvent for exhaustive extraction of herbs (Wang and Weller, 2006). Literature indicated that extraction of herbs such as ginseng by conventional methods takes several hours (12 h) (Kwon et al., 2003). Also, the usage of organic solvents could reach up to 100 mL per g of sample for the extraction of leaves of *Iochroma gesneriodes* (Kaufmann et al., 2001).

Therefore, novel green methods of extraction such as microwave-assisted extraction (MAE) can be utilized to extract the plant to meet the rising demand for extracts for commercial application. In this non-conventional method, electromagnetic radiation was applied to enhance the kinetics of extraction, and could also improve the quality of extracts. Microwave energy is absorbed by the polar compounds according to their dielectric constant and is dissipated as heat to the surroundings. Thus the heating of the specific metabolites can be targeted in the plant samples for better extraction (Eskilsson and Bjorklund, 2000). In past, the yield of secondary metabolites from various herbs has also improved by using the MAE (Chan et al., 2011; Zhang et al., 2011; Arya et al., 2022). For example, the recovery of triterpenoid saponins from *Ganoderma artrum* enhanced significantly for 15 min of MAE at 800W and 70°C as compared to conventional methods of extraction (Chen et al., 2007). Similarly, MAE of *Radix astragali* at 700W and 70°C for 15 min enhanced the concentration of astragalosides-I to more than twice as compared to the maceration process (Yan et al., 2010).

A broad investigation has been designed and executed to extract the selected herb by green methods such as microwave-assisted extraction along with conventional techniques like maceration. Further, the extraction conditions in MAE are also optimized by using box-behnken design coupled response surface methodology. The extracts were also analyzed by GC-MS to confirm the presence of various secondary metabolites. Also, the anti-Alzheimer’s potential of different extracts was assessed on the various behavioral and biochemical parameters along with acetylcholinesterase (AChE) inhibitory potential.

## 2 Materials and methods

### 2.1 Plant sample and solvents

Dried roots of *Nardostachys jatamansi* (NJ) were procured from the market near Hisar, Haryana, India, and identified by a botanist before extraction by different techniques. The chemicals and solvents utilized for extraction and other investigations were of AR grade. Standard drugs like Donpezil and scopolamine were purchased from Sigma Aldrich, USA.

### 2.2 Extraction by maceration

The conventional extraction method, maceration was chosen to carry out the extraction of the selected herb. Briefly, in maceration, the plant part taken was coarsely powdered, sieved (40 size), and transferred to a conical flask containing ethanol: water (80:20) as solvent. After keeping it undisturbed for eight days, the sample solution was then filtered and kept in a water bath to get a concentrated product. Later, the extract yield was calculated and the extract was kept in an airtight container until further analysis was performed (Devgan et al., 2013).

### 2.3 Microwave-assisted extraction (MAE)

Microwave synthesis apparatus (CEM, Model: Discover system, 908010, Matthewis, NC; USA) was used to perform the microwave treatment on the powdered roots. Throughout the study, the solid/solvent ratio (1:15) was kept constant for hydroalcoholic solvent (80:20) to carry out the novel extraction procedure. About 0.4 g of sample powder was irradiated using varying operating conditions of microwave power, temperature, and irradiation period as proposed by the software used. During the experiments, the microwave power and the temperature were adjusted automatically to a particular set of conditions and displayed on the screen of the instrument. After the completion of the extraction process, the extract was kept at room temperature and filtered after centrifuging it for 5 min at 2000 rpm. Subsequently, a rotary evaporator was used to concentrate the extract and weighed it for percentage yield (w/w) calculation (Mittal and Nanda, 2017).

### 2.4 Experimental design

In this study, various parameters such as microwave power (X_1_: 100-300 watts), temperature (X_2_: 50-90°C), and irradiation time (X_3_: 2-20 _min_) were optimized using box-behkhen design (BBD) (**Table 1**). The design expert software (7.0.3, Statease Inc, Minneapolis, USA) was used for experimental design which suggested 17 trial runs and was carried out accordingly. Responses were expressed in terms of dependent variables like extraction yield (Y_1_), total phenolic content (TPC; Y_2_), and total flavonoid content (TFC; Y_3_) to study the effect of independent factors (Filip et al., 2017).

**Table 1:** Coded levels with actual values of different variables for experimental design.

Variables	Unit	Coded level with actual value		
		-1	0	+1
**Microwave power (X_1_)**	Watt	100	200	300
**Temperature (X_2_)**	°C	50	70	90
**Time (X_3_)**	min	2	11	20

#### 2.4.1 Total phenolic content (TPC)

The TPC for obtained extracts was measured using the Folin-Ciocalteu method. Shortly, in the method adopted, the extract was dissolved in methanol (10mg/ml). Then, 1.5 ml Folin-Ciocalteu reagent was added to the above-prepared solution. After 10 min, 1.5ml of 25% aqueous Na_2_CO_3_ was mixed and the mixture was stirred. Later, the mixture was incubated in a water bath for 30 min at 45°C. UV- visible spectrophotometer (UV-1800, Shimadzu Scientific Instruments Private Limited) was used to analyze the resultant samples against the blank sample at 760nm. To obtain the straight-line equation, a standard solution of Gallic acid was prepared and analyzed for the concentrations ranging between 50-250 μg/ml. To calculate the TPC quantitatively, readings for absorbance were taken in triplicate and expressed as mg GAE/g of the extract while the fallouts were stated as mean ± standard deviation (S.D.) (Sharma and Singh, 2012).

#### 2.4.2 Total flavonoid content (TFC)

To evaluate the total flavonoid content (TFC) aluminum chloride method was used. A required amount of plant extract was mixed with methanol (10 mg/mL). Then, 1.5 mL 2% aluminum chloride solution was prepared and 1.5 mL was added to the mixture and kept aside for 1 h at room temperature. Later, the absorbance was estimated with the help of a UV-visible spectrophotometer at a wavelength of 415 nm against the blank. The TFC values were expressed in mg rutin equivalent/g of extract. The observations were observed in the triad and shown as mean ± standard deviation (S.D.) (Sharma and Singh, 2012).

### 2.5 Scanning electron microscopy (SEM)

SEM was used to analyze the possible modification in the microstructure of powdered plant samples before and after the extraction process. To prepare the samples for SEM, the residuals obtained after the extraction process were dried under vacuum at 40°-50°C for at least 2 h and coated properly with gold before microscopical analysis (Zhou and Liu, 2006; Zhang et al., 2008).

### 2.6 GC-MS analysis

The analysis of extracts obtained by chosen extraction methods (maceration and MAE) was carried out by Gas chromatography coupled with mass spectra (Shimadzu QP-2010 having Thermal Desorption System TD 20; fitted with MS capillary column). Electron ionization system with the chosen parameters,70 eV energy; helium as carrier gas; injector temperature at 260°C with 1.5 mL/min flow rate were the chosen parameters used for GC-MS analysis. Initially, the column temperature was 70°C for 2 min and later accelerated to 150°C at a rate of 3°C/min for 10 min and lastly increased to 250°C at the rate of 4°C/min. Then, the required volume of 1 μL sample was injected manually in a split manner to obtain the chromatogram and identify the constituents present (Razack et al., 2015).

### 2.7 *In-vivo* anti-alzheimer’s evaluation

#### 2.7.1 Animals

Institutional Animal Ethical Committee, IAEC, MDU, Rohtak granted approval for our research protocol presented against vide reference number 1767/RE/S/14/CPCSEA/CAH/76-85 dated 26.02.2021. The mice were Swiss albino weighing between 25-30g, procured from an aseptic animal house, Lala Lajpat Rai University of Veterinary and Animal Sciences, (LLRU-VAS), Hisar, India. Polypropylene cages were used to retain the animals throughout the study. All the mice had an appropriate approach to water *ad-libitum* along with the dried feed and 12 h light/dark sequence at 25 ± 2°C. House conditions were set and sustained consistently in reference to the CPCSEA guidelines.

#### 2.7.2 Experimental protocol

Swiss albino mice were equally divided into six groups (n=5) and provided with dry feed along with water for seven days prior to dosing. The first group, the control, was given normal saline. Scopolamine and donepezil in doses of 0.4mg/kg and 3mg/kg, i.p were administered to the 2^nd^and 3^rd^group respectively while the animals of groups fourth and fifth were administered orally with various extracts prepared (200 mg/kg) for the next fourteen days. On the 15^th^ day of dosing, test group animals were injected with scopolamine (0.4 mg/kg, i.p.) to evaluate the protective effect of different extracts against amnesia induced by scopolamine with the help of below-mentioned parameters.

##### 2.7.2.1 Evaluation of behavioral parameters

The behavioral studies were performed using (i) an elevated plus maze and (ii) passive avoidance apparatus to analyze the learning and memory potential found in various groups of mice. As the name suggested plus maze is a **+** (plus) shaped wooden block consisting of open and closed arms in counter directions and is placed at an elevated height of 50 cm from the floor. In this model, one mouse at a time was placed towards the open arm of the instrument and its movement was observed for 5 min to estimate transfer latency (TL) i.e., the time the mouse takes to move inside the covered arm (Vasudevan and Parle, 2006). A passive avoidance test is commonly employed to describe the investigational practice in particular behavioral suppression to sidestep noxious circumstances, learned by the chosen animals. In this apparatus, 60V electric current at a frequency of 1Hz for 0.5sec was applied to the steel grid on the floor to determine the step-down latency (SDL), i.e. the time taken by the mice to reach the wooden floor from the steel grid floor). During training, each mouse was kept on the wooden floor and an electric current was passed into the steel grid floor. The time that the mouse takes to come back to the floor deprived of current i.e., the wooden floor, from the steel grid floor was noted. All mice were trained for 24 h and SDL was recorded for five minutes to evaluate passive avoidance behavior characteristics in mice (Joshi and Parle, 2006).

##### 2.7.2.2 Estimation of biochemical parameters

On the 17th day of the experimental procedure, complete mice brain was isolated from each group and cleaned thoroughly with standard ice cold saline. The phosphate buffer with pH 7.4 was used to homogenize the brain samples and centrifuged at 10000 rpm for 15min. The brain homogenates were further analyzed for several biochemical parameters. Catalase activity was carried out according to Aebi’s method (Aebi, 1974). To carry out the process, 0.1 mM phosphate buffer (pH 7.4) and 1 mL of 30 mM hydrogen peroxide were mixed with 100 μL brain homogenate, and this reaction mixture was analyzed at a wavelength of 240 nm using UV- spectrophotometer. Absorbance variation was noted and stated as nmol of H_2_O_2_ consumed/min/mg/protein.

Glutathione activity was performed using Ellman’s method (Ellman, 1959). To perform this, an equal quantity of brain homogenate was dissolved in an equal quantity of 10% of trichloroacetic acid. Then, for 15 min centrifugation was carried out to isolate the proteins present in the blend. After centrifugation, 0.01 mL of supernatant was dissolved in 2 mL phosphate buffer having pH 8.4 and with vigorous stirring added 0.5 mL 5’5-dithiobis (2-nitrobenzoic acid) to water. The absorbance of the reaction mix was noted using UV- spectrophotometer at 412 nm within 15 min and dropped glutathione concentration was expressed in μmol/mg tissue. Brain homogenate is evaluated to evaluate the content of nitric oxide present using Griess Reagent and calculated as total nitrate/nitrite (NOx) (Lieben and Blokland, 2005). The chemical reaction of nitrate to nitrite is the result of vanadium trichloride. Briefly, in this mechanism, sulfanilamide diazotization is responsible for the formation of chromophores as the binding of acidified nitrite with N-(1-naphthyl) ethylenediamine lead to the formation of a colored derivative of azo which was later analyzed using spectrophotometer at a wavelength 540 nm and stated as μmol/mg tissue. In various groups, mice brain homogenates were used to determine the concentration of superoxide dismutase (SOD). To perform this procedure, brain homogenate was mixed with n-butanol with continuous stirring. The obtained reaction mix was kept uninterrupted for some time and after 15 min of centrifugation, n-butanol was separated. UV-Visible spectrophotometer at a wavelength of 560 nm was employed to take the absorbance of the solution (Kakkar et al., 1984).

##### 2.7.2.3 Acetylcholinesterase (AChE) inhibitory potential

Ellman’s method was used to evaluate the inhibitory potential of AChE in brain homogenates for extracts obtained by maceration and optimized microwave-assisted extraction (Ellman et al., 1961). To carry out this method, brain homogenate, phosphate buffer (0.1M; pH 8) and dithiobisnitro-benzoic acid (0.01M) were mixed and the reaction mixture was incubated at room temperature for the next 5 min. Acetylthiocholine iodide substrate was added to the mixture and the absorbance was recorded at 412 nm.

### 2.8 Statistical analysis

Graphpad Prism 9.0 was applied to evaluate the data significance using analysis of variance (ANOVA). The results observed were taken in triplicate throughout the study.

## 3 Results and discussion

*Nardostachys jatamansi* (NJ), commonly known as ‘*Jatamansi’* possesses an exclusive role in Ayurvedic medicinal system and is conventionally consumed in preventing mental illness and also to improve learning and memory power (Dhiman and Bhattacharya, 2020). In the preparation of traditional herbal formulations, the powdered rhizomes and the roots of the plant, *Nardostachys jatamansi*, were utilized. The extraction was generally carried out using various conventional techniques such as maceration and percolation. But, considering the remedial and economical worth of the selected plant together with the global demand these traditional methods do not seem to be sufficient. Also, the limitations associated with conventional methods of extraction made us implement one of the modern, time-saving, and green extraction methods i.e., Microwave Assisted Extraction (MAE). Various extraction parameters in novel extraction techniques were also optimized using CCD coupled with response surface methodology.

Our investigation intended to comparatively assess the various extracts for probable enhancement in the recovery of secondary metabolites along with improved pharmacological potential. Further, it is well established that the collection of plant samples from the correct source dictates the quality and quantity of plant actives (Sutivisedsak et al., 2010). Moreover, the *Jatamansi* roots are highly confused with the Valerian species, thus the procured roots of the selected plant were first identified and authenticated by a botanist (Mabberley and Noltie, 2014). The morphological characters of the collected roots were compared with the standard sample present in the Raw Material Herbarium and Museum by Dr. Sunita Garg, Scientist, CSIR, NISCAIR, Delhi. On the basis of a comparative morphological study, the collected sample of NJ roots was confirmed as *Nardostachys jatamansi* (D. Don) DC vide reference number NISCAIR/RHMD/Consult/2018/3157-06-2 dated 19/03/2018. After authentication of collected NJ roots, these were powdered coarsely and extracted with conventional (maceration) and non-conventional (microwave-assisted extraction) methods of extraction. The extraction yield (%) for the maceration process was found to be 5.35 ± 0.14% w/w and this extract is termed MCNJ in this manuscript.

### 3.1 Microwave-assisted extraction

Among various promising green and effective modern technologies of extraction, microwave-assisted extraction is quite prevalent and works with electromagnetic radiation. In this method, the solvent and the plant material are heated up rapidly by high-speed energy radiations which leads to the efficient and time-saving extraction of constituents (Wang and Weller, 2006). Improved dipole rotation accompanied by better ionic conduction in the solvent particles enables the efficient separation of desired molecules from the plant matrix (Wang and Weller, 2006; Chemat et al., 2009; Chemat et al., 2012). Numerous factors such as microwave power, solvent nature and concentration, time of irradiation, solid-to-solvent ratio, temperature for extraction, and particle size need to be optimized as these parameters can alter the phytoconstituent yield and extraction efficiency significantly (Wang and Weller, 2006). In this study, for the microwave extraction of the selected herb, based on some preliminary investigations, hydroalcoholic solvent (80:20) was selected. The literature also suggests that polar solvents like water and ethanol are best suited for microwave irradiation. The addition of water to the ethanol improves the dielectric constant of the mixture to some extent (Alara et al., 2018). Thus as the water is added (upto20%) to the organic solvent, it enhances the polarity indices of the solvent and resulted in improved microwave energy absorption along with the solubilization of plant actives. Further, it also elevated the temperature of the inner wall of the particles of plant sample and leads to the cell wall rupture, and accelerated the phytoconstituents recovery (Eskilsson and Bjorklund, 2000; Chan et al., 2011; Bouras et al., 2015; Moreira et al., 2017). Likewise, solvent-to-solid ratio is also a critical factor in the extraction of plant samples by MAE. A sufficient volume of solvent is required for the absorption of microwaves and also to solubilize the bio-actives (Wang et al., 2008; Zhang and Yang, 2009). Therefore the ratio (15:1) was also selected on the basis of the results of some initial experiments. Further increasing the amount of solvent did not improve the extract, or the TPC and TFC yields. The microwave energy dissipated in heating the higher amount of solvent and did not impact the plant particles (Gao et al., 2007). Consequently, the more parameters which can affect the concentration of plant actives along with extraction kinetics are also optimized using box-behnken design (BBD).

### 3.2 Model fitting for MAE

The effect of various selected independent variables like microwave power, temperature, and irradiation time on different dependent variables (EY, TPC, and TFC) was calculated using BBD coupled response surface methodology Further the design proposed a run of a total of 17 experiments which were carried out accordingly and the observations were represented in **Table 2**. Also, the statistical analysis of the data was carried out and it confirmed the significance of all the linear (X_1_, X_2,_ and X_3_), interactive (X_1_X_2_, X_2_X_3_), and quadric terms ( ${\text{X}}_{1}^{2}$ , ${\text{X}}_{3}^{2}$ and ${\text{X}}_{3}^{2}$ ) for EY, TPC, and TFC (**Table 3**). To verify, if the model is significant or not statically, an analysis of variance was performed on the experimental data obtained. The higher F value indicated by the developed model for Y_1_ (~99), Y_2_(~980), andY_3_ (~145) proved that the model chosen was significant. Also, the probability value was found lower, i.e., p<0.0001; signifying the extremely significant behavior of the model while the regression equation can describe the response difference clearly. The degree of fitness was estimated by R^2^ values of EY, TPC, and TFC (0.99) and basically it is the explained to total variance ratio. The vicinity of R^2^ to unity showed the well-fitted developed model in reference to the actual data. Similarly, predicted R^2^ values of (EY 0.91, TPC 0.99, and TFC 0.96) presented a good correlation of the selected response with the independent variables. Moreover, a small value of the coefficient of variance of EY (~4), TPC (1.42), and TFC (4.2) validates the dependability of data and accuracy to noise proportion justifying the developed model fitness to direct the design space. Further on applying multiple regression analysis, a second-order polynomial mathematical equation was also derived. The magnitude of the effects of different variables on the responses like EY (Y_1_), TPC (Y_2_), and TFC (Y_3_) were also represented by Equations 1,2,3.

**Table 2:** Actual and Predicted values for extraction yield, TPC and TFC for different parameters (MAE).

Std.	Run	Power(watt) (X_1_)	Temp.(°C)(X_2_)	Time(min)(X_3_)	EY (%w/w)		TPC (mg of GAE/g of extract)		TFC (mg of RUE/g of extract)	
					Actual Yield (w/w)	Predicted Yield (w/w)	Actual Yield (w/w)	Predicted Yield (w/w)	Actual Yield (w/w)	Predicted Yield (w/w)
**1**	1	100	50	11	1.9	2.37	9.5	9.57	2.16	2.96
**4**	2	300	90	11	13.01	13.14	60.98	60.91	29.2	29.20
**6**	3	300	70	2	14.00	14.69	68.09	68.26	31.01	32.01
**13**	4	200	70	11	13.01	13.79	64	64.10	32	32.00
**15**	5	200	70	11	13.08	13.79	65	64.10	32.9	32.90
**14**	6	200	70	11	13.07	13.79	65.3	65.40	30.89	31.94
**2**	7	300	50	11	13.05	14.73	73.46	73.57	37.5	37.48
**11**	8	200	50	20	13	12.93	55.71	55.81	30	29.68
**7**	9	100	70	20	10	10.20	30.92	30.75	13.48	14.17
**8**	10	300	70	20	12.65	13.05	55.01	55.00	24.12	24.46
**5**	11	100	70	2	6	5.60	18.89	18.90	5.5	5.16
**9**	12	200	50	2	12.56	13.08	59.22	59.14	28.96	29.68
**3**	13	100	90	11	13.23	13.56	51	51.09	28	28.02
**16**	14	200	70	11	13.89	13.79	63	64.10	30.41	31.94
**10**	15	200	90	2	16.18	16.25	71.14	71.04	37.05	37.37
**17**	16	200	70	11	13.99	13.79	63.21	64.10	33.5	31.94
**12**	17	200	90	20	19.88	19.36	72.89	72.97	40.24	39.52

**Table 3:** ANOVA applied on obtained data for significance and suitability of design and different variables.

Extraction yield (EY)						TPC					TFC				
Source	Sum of squares	Df	Mean	F	p-value	Sum of squares	Df	Mean	F	p-value	Sum of squares	Df	Mean	F	p-value
**Model**	235.16	9	26.13	99.18	< 0.0001***	5521.96	9	613.55	980.85	< 0.0001***	1775.34	9	197.26	145.21	< 0.0001***
**X_1_**	71.22	1	71.22	270.35	< 0.0001***	2709.58	1	2709.58	4331.67	< 0.0001***	664.12	1	664.12	488.88	< 0.0001***
**X_2_**	46.03	1	46.03	174.73	< 0.0001***	422.24	1	422.24	675.02	< 0.0001***	153.74	1	153.74	113.17	< 0.0001***
**X_3_**	4.35	1	4.35	16.52	0.0048***	0.9870	1	0.9870	1.58	0.2494	2.33	1	2.33	1.72	0.2314
**X_1_ X_2_**	40.77	1	40.77	154.75	< 0.0001***	728.46	1	728.46	1164.55	< 0.0001***	277.89	1	277.89	204.56	<0.0001***
**X_1_X_3_**	9.73	1	9.73	36.95	0.0005***	157.63	1	157.63	251.99	< 0.0001***	62.96	1	62.96	46.35	0.0003***
**X_2_ X_3_**	2.66	1	2.66	10.09	0.0156*	6.92	1	6.92	11.06	0.0127*	1.16	1	1.16	0.8507	0.3870
**x_1_^2^**	57.00	1	57.00	216.36	< 0.0001***	1431.68	1	1431.68	2288.75	< 0.0001***	547.68	1	547.68	403.16	< 0.0001***
**x_2_^2^**	2.98	1	2.98	11.30	0.0121*	39.75	1	39.75	63.55	< 0.0001***	63.39	1	63.39	46.66	0.0002***
**x_3_^2^**	2.55	1	2.55	9.68	0.0170*	24.96	1	24.96	39.90	0.0004***	13.01	1	13.01	9.57	0.0175*
**Residual**	1.84	7	0.2634			4.38	7	0.6255			9.51	7	1.36		
**Lack of Fit**	1.20	3	0.3985	2.46	0.2026	0.1166	3	0.0389	0.0365	0.9893	2.71	3	0.9023	0.5306	0.6851
**Pure Error**	0.6485	4	0.1621			4.26	4	1.07			6.80	4	1.70		
**C.V. %**	4.00					1.42					4.23				
**R^2^**	0.9922					0.9992					0.9947				
**Adjusted (R^2^)**	0.9822					0.9982					0.9878				
**Predicted (R^2^)**	0.9150					0.9985					0.9698				
**Adequate Precision**	43.1435					105.173					41.3223				

p-value<0.05 indicates the significance of data (*significant; ***highly significant).

Equation 1


$$(Y_{1})=235.16+71.22X_{1}+46.03X_{2}+4.35X_{3}+40.77X_{1}X_{2}+9.73X_{1}X_{3}+2.66X_{2}X_{3}+57.00X_{1}^{2}+2.98X_{2}^{2}+2.55X_{3}^{2}$$

Equation 2


$$(Y_{2})=5521.96+2709.58X_{1}+422.24X_{2}+0.9870X_{3}+728.46X_{1}X_{2}+157.63X_{1}X_{3}+6.92X_{2}X_{3}+1431.68X_{1}^{2}+39.75X_{2}^{2}+24.96X_{3}^{2}$$

Equation 3


$$(Y_{3})=1775.34+664.12X_{1}+153.74X_{2}+2.33X_{3}+277.89X_{1}X_{2}+62.96X_{1}X_{3}+1.16X_{2}X_{3}+547.68X_{1}^{2}+63.39X_{2}^{2}+13.01X_{3}^{2}$$

#### 3.2.1 Diagnostic of model adequacy

**Figure 1:**
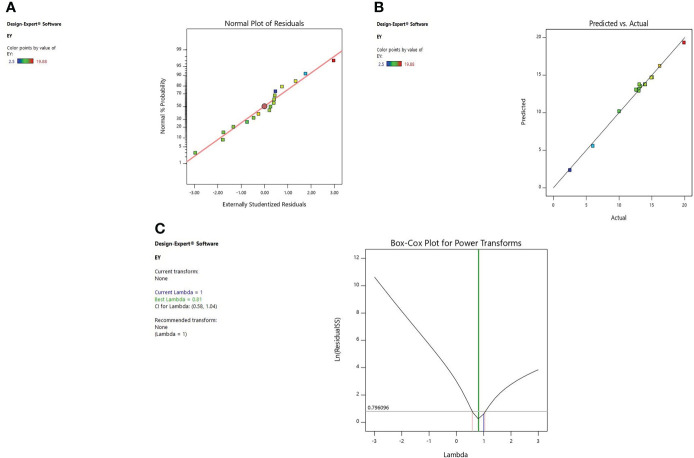
Diagnostic plot of Extraction yield (EY); **(A)** Normal % probability versus internal residual, **(B)** Actual versus predicted values, **(C)** Box-cox plot.

**Figure 2:**
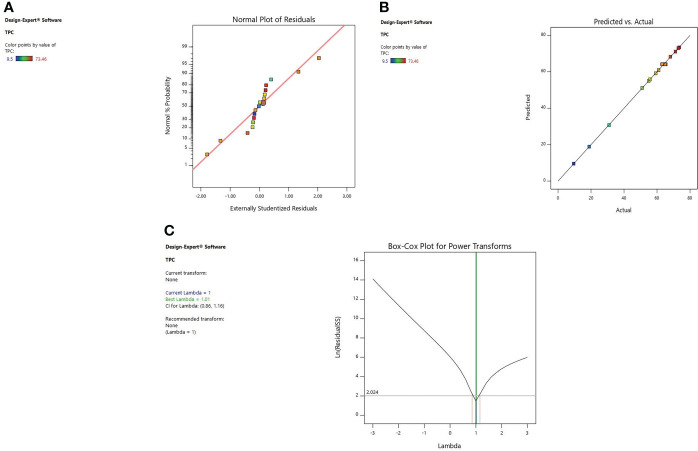
Diagnostic plot of TPC; **(A)** Normal % probability versus internal residual, **(B)** Actual versus predicted values, **(C)** Box-cox plot.

**Figure 3:**
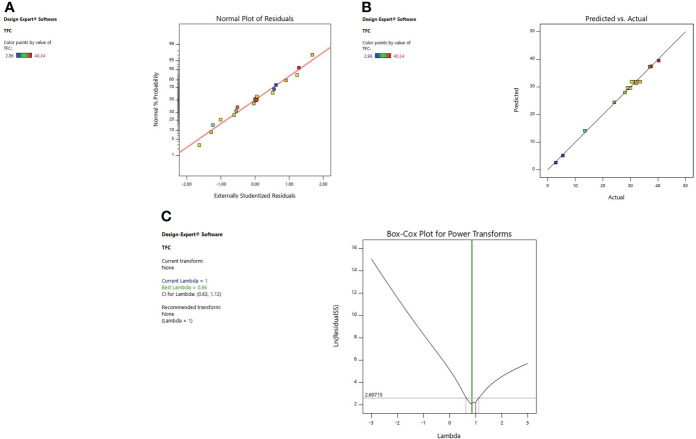
Diagnostic plot of TFC; **(A)** Normal % probability versus internal residual, **(B)** Actual versus predicted values, **(C)** Box-cox plot.

Diagnostic plots were also used to estimate the adequacy of the developed model. The residual plot between normal percentage probability and internal residual was found normal without any significant variance proving the developed model fitness (**Figures 1A**, **2A**, **3A**). The plot between actual and predicted values evidently depicts that predicted values are situated close to a straight line and are in conformity with real values for EY, TPC, and TFC (**Figures 1B**, **2B**, **3B**). Also, the λ value of the obtained data is near to 1 as indicated in box-cox plot (**Figures 1C**, **2C**, **3C**) and no power transform is required for the dependent variables (EY, TPC, and TFC).

#### 3.2.2 Response surface analysis

**Figure 4:**
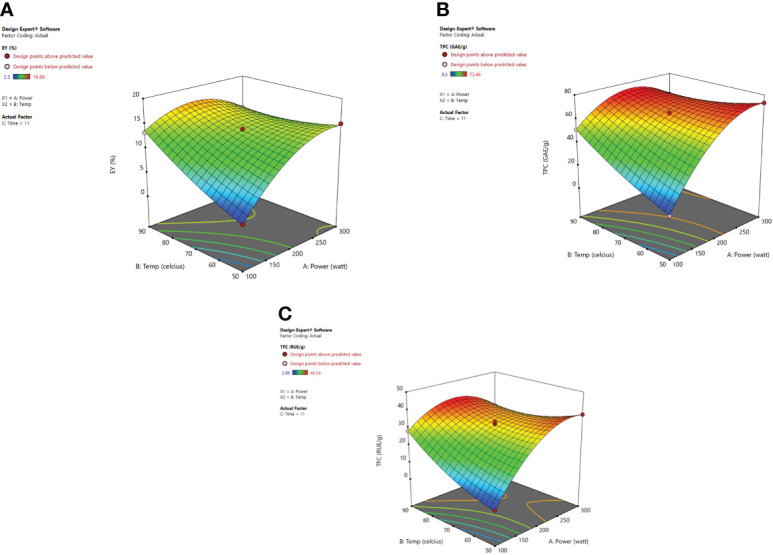
3-D diagrams indicating the effect of different variables (X_1_, X_2_) on response, **(A)** EY (Y_1_), **(B)** TPC (Y_2_), **(C)** TFC (Y_3_) in MAE.

Response surface analysis **Figure 4** revealed that microwave power (X_1_) along with the temperature (X_2_) at a constant time factor (11 min) impart an encouraging significant effect on extraction yield, TPC, and TFC. Extraction yield has been influenced by microwave power significantly. A higher value for EY was observed at 200 W and after that, a decrease was noticed in the extraction yields up to 300 watts. A decrease in extraction yield has been observed because of the thermal degradation of phytoconstituents for higher values of microwave power at which the potent heat produced can reduce the number/quantity of phenolic components present in plant material (Spigno and De Faveri, 2009; Dahmoune et al., 2014; Xiong et al., 2016 **).** Microwave power increases as the irradiation time is elevated due to which the heat produced within the cellular part of the plant and obtained extract causes a faster release of phytoconstituents. In a dispersion medium, the dipole rotation along with the ionic conduction of molecular particles generally relies on heat intensity. The dipoles present in solvent go through an oscillatory motion and later are arranged in the electronic field direction which happens at a higher rate so as to cause heat generation within the medium (Eskilsson and Bjorklund, 2000). Furthermore, the molecules in the solvent start to dissociate into ions also known as charged particles, and the increased flow rate of ions is detected in the vessel due to the applied electronic field. The excessive ion movement results in an increased ionic collision and friction energy is generated which accelerates the temperature in the medium (Gfrerer and Lankmayr, 2005; Nadagouda et al., 2011; Ahmad et al., 2017). The moisture existing in the plant material is vaporized due to the heat and the cracks on the cell walls of the plants are produced because of the thermal effect of microwaves. These cracks cause solvent penetration within the cells which in turn leads to solubilization and oozing out of the active phytoconstituents in the surrounding liquid (Rostagno et al., 2009). The concentration of the phytoconstituents was not affected by the microwave power after the total exhaustion of plant material and if further exposure of power is given to the plant it may degrade the chemical structure of polyphenolic compounds and would lead to decreased extraction yield (Hao et al., 2002). Xiong et al. (2016) developed the extraction conditions for efficient microwave-assisted extraction of *Lotus plumue* using a central composite design of RSM and selected 200 W as optimized microwave power.

**Figure 5:**
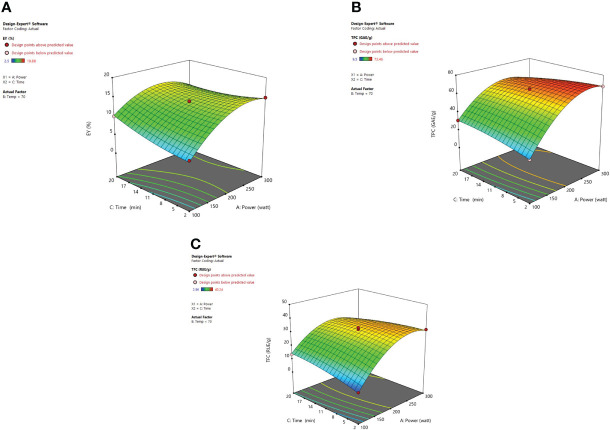
3-D diagrams indicating the effect of different variables (X_1_, X_3_) on response, **(A)** EY (Y_1_), **(B)** TPC (Y_2_), **(C)** TFC (Y_3_) in MAE.

The irradiation time of microwaves is also an important factor in the extraction of desired phytoconstituents from herbal plants. From response surface analysis **Figure 5**, it was revealed that microwave power (X_1_) along with the time (X_3_) at a fixed temperature of 70°C imparts an encouraging effect on extraction yield, TPC, and TFC. At 200W, it was observed that the recovery of phenolic compounds and extraction yield of the extract increased steadily up to 20 minutes, and further a decrease in the extraction yield was noticed beyond 20 minutes. Also, the higher power applied for a stretched irradiation time can affect the chemical structure of the targeted compounds adversely (Xiong et al., 2016). In various MAE studies, it has been indicated that both the factors (i) high microwave power and (ii) prolonged irradiation time can degrade the plant metabolites significantly (Gfrerer and Lankmayr, 2005; Rostagno et al., 2009; Nadagouda et al., 2011; Ahmad et al., 2017). If the solution resists the electrophoretic movement of ions, then it would lead to fast heating of sample material within the solvent mix. Because of this rapid increase of temperature in the solvent mix, the cell walls of the plant matrix got ruptured owing to boosted cavitations and turbulence possessions that results in enhanced mass transfer (Hao et al., 2002; Routray and Orsat, 2014; Fernandez-Pastor et al., 2017). After these observed parameters, no substantial alteration was detected in the response factor (EY). Moreover, in the MAE technique, if the plant material is exposed to irradiations for a prolonged period, the concentration of desired compounds could decrease due to overheating of the plant sample at elevated temperatures. Besides, literature corroborates the same that when irradiation time increases continuously, then after a certain time period extraction yield begins to drop. Extended time duration of microwave irradiation exposure can degrade the active constituents present in the matrix and also reduce the extraction yields considerably (Qiu et al., 2012; Ahmad et al., 2017).

**Figure 6:**
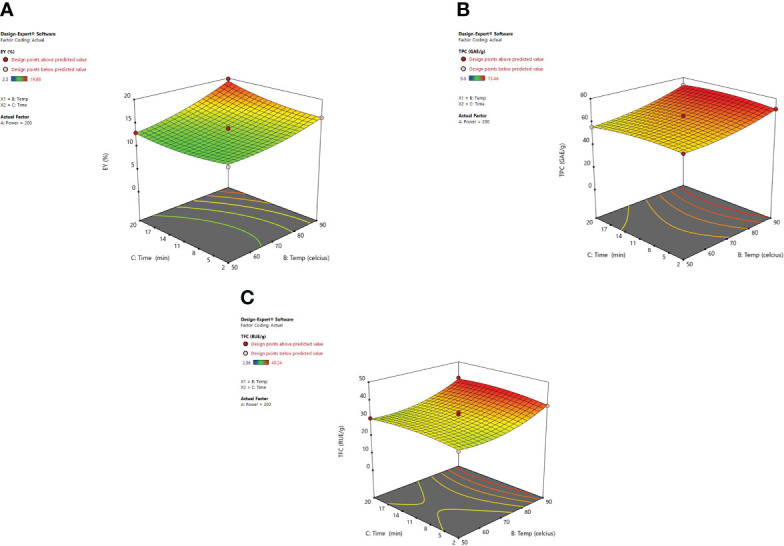
3-D diagrams indicating the effect of different variables (X_2_, X_3_) on response, **(A)** EY (Y_1_), **(B)** TPC (Y_2_), **(C)** TFC (Y_3_) in MAE.

Another imperative factor is the temperature, which influences the extraction yield while using MAE. **Figure 6** shows the significant effect of temperature **(**X_2_) and irradiation time (X_3_) at a constant microwave power of 200 W on the EY, TPC, and TFC. As heating enhanced the diffusivity of the solvent mixture in the plant material so solubility for the recovery of phenolic content from plant material was also improved (Spigno and De Faveri, 2009; Chew et al., 2011; Dahmoune et al., 2014; Alara et al., 2018). Higher temperatures were found to affect the extraction yields positively though cannot be enhanced indefinitely. Various studies showed that 60-80°C temperature range is quite suitable for the recovery of phytoconstituents using microwave-assisted extraction. (Chan et al., 2011; Shang et al., 2016). Ahmad et al. (2017) demonstrated that when very high temperatures are used to carry out MAE then the sample matrix can be degraded and lead to alteration in the structure of desired compounds (Gfrerer and Lankmayr, 2005). Also, Routray and Orsat (2012) observed an increase in extraction efficiency with the elevated temperatures until an optimum temperature was attained. Later, efficiency begins to decrease with the continual increase in temperature, this occurs as a consequence of the selected ideal extraction temperature which is directly associated with the stability and yield of the targeted compound(s). To an extent, the factors such as irradiation time and microwave power influence each other. A low to moderate power combination with longer irradiation exposure is usually selected to optimize the MAE procedure. Our results were clearly in correlation with the study of Gandhi et al. (2019) and Alara et al. (2018). According to their studies, an increase in extraction temperature up to 90°C not only increased the extraction efficiency but also the extraction yield of betulic acid was enhanced. In MAE closed system, temperature escalation results in the vapor pressure rise so as to improve the efficiency of the extract because the chemical compounds are desorbed from the sample matrix. The rise in temperature leads to a decrease in surface tension along with the lowering of solvent viscosity and that results in improved sample wetting and solvent diffusion power respectively. With the increase in temperature the solute starts releasing from the active sites of the matrix and as a result, improved extraction efficiency is achieved (Eskilsson and Bjorklund, 2000). The energy provided to the plant matrix from microwave power is converted into heat energy and hence the temperature in the vessel can be controlled (Mandal et al., 2007; Khajeh et al., 2010; Li et al., 2010).

#### 3.2.3 Optimization and validation of the predictive model

In this current study, various parameters were optimized for MAE using BBD-coupled RSM to attain maximum extraction yield (%), TPC, and TFC. A numerical optimization method was adopted and optimized extraction conditions for MAE were predicted (microwave power 187.04 W, temperature 90°C, and irradiation time 20 min) to achieve the maximum EY, TPC, and TFC. The root of the NJ was also extracted at the optimized conditions of MAE (extract termed as OMNJ) and all responses were observed to lie within 95% confidence and thus validated the established model. The comparative extraction conditions and responses in different methods (OMNJ and MCNJ) are presented in **Table 4**.

**Table 4:** A comparative assessment for the various extracts of NJ for EY, TPC and TFC.

Extract	Power(watt)	Temperature(^o^C)	Time of extraction	EY (%, w/w)	TPC (mg of GAE/g of extract)	TFC(mg of RUE/g of extract)
**OMNJ**	187.04	90	20 min	19.52±0.5	72.83±1.1	39.21±0.7
**MCNJ**		(25-30)	8 days	5.35±0.14	32.13±0.55	21.7±0.85

### 3.3 Scanning Electron Microscopy (SEM)

**Figure 7:**
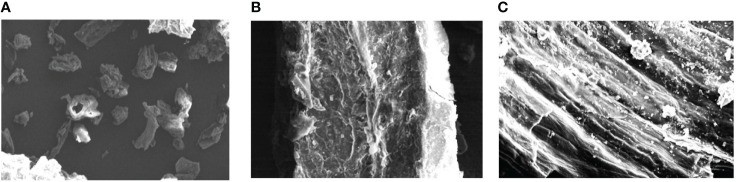
SEM of different samples **(A)** Unprocessed sample **(B)** after maceration **(C)** MAE.

To confirm the structural changes in the powdered plant samples after the extraction with different methods (maceration and MAE), scanning electron microscopy (SEM) was generally employed (Liu et al., 2006; Lucchesi et al., 2007). The powder of roots of selected medicinal herb obtained after drying of the marc (after maceration and MAE) was also subjected to SEM. The intact powder of the plant sample (without extraction) was also analyzed by SEM to further compare the micrographs of different samples. The photomicrograph of untreated powder of selected herb showed uniform and consistent cellular surface of particles (**Figure 7A**). Likewise, on maceration treatment, the particles lost uniformity in shape and the surface became somewhat flat (**Figure 7B**). Plant tissues were significantly disturbed and crumbled on treatment with microwave radiations (**Figure 7C**). Absorption of microwave energy by moisture present in the plant particles and further vaporization of this created high pressure inside the sample which causes the disruption and crumbling of plant samples (Rostagno et al., 2009). Similar results were also reported by the previous researchers for the samples of *Erigeron breviscapus* and tobacco leaves, where the microwave treatment significantly causes the change in the microstructure of the plant samples (Zhou and Liu, 2006; Gao et al., 2007).

### 3.4 GC-MS analysis

**Figure 8:**
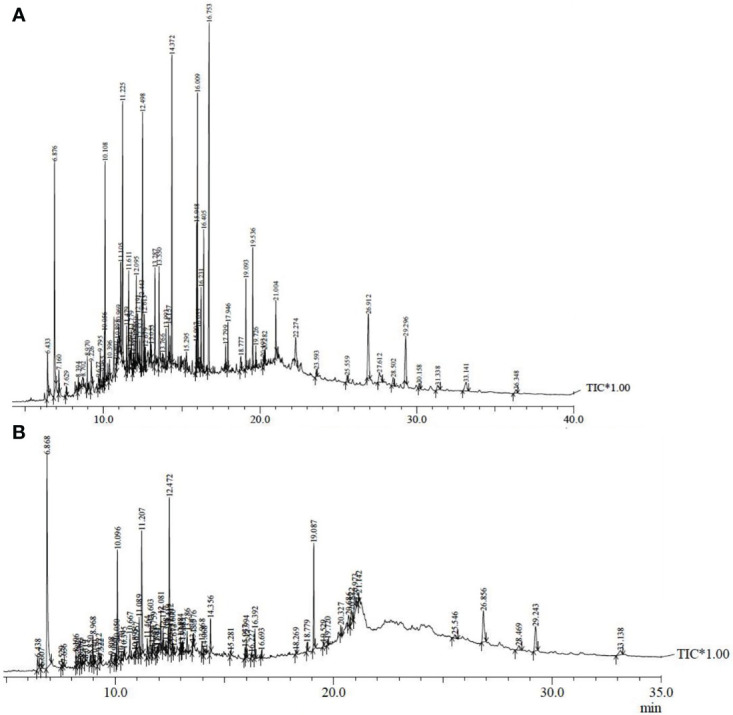
GC-MS chromatogram for different extracts **(A)** MCNJ **(B)** OMNJ.

A literature study revealed that MAE significantly enhanced the concentration of secondary metabolites from plants (MAE review reference). Hence the extract obtained at optimized conditions of green approach (OMNJ) was also subjected to GC-MS analysis for the tentative confirmation of plant actives present in it. Retention time (R_T_) and percentage (%) area metabolites is also compared in the two extracts (**Table 5**). The GC-MS chromatogram of MCNJ and OMNJ is also presented in **Figure 8**.

**Table 5:** GC-MS analysis of NJ extracts for different secondary metabolites.

	MCNJ		OMNJ	
Chemical Constituents	R_T_ (min)	Area (%)	R_T_ (min)	Area (%)
Spirojatamol	10.108	4.29	10.096	8.0
Jatamansone	11.225	4.88	11.207	7.86
Valerenal	11.611	2.50	11.603	3.02
Globulol	12.498	5.52	12.472	6.71
Nootkatone	12.679	0.39	12.673	0.40
Valeric acid	13.550	2.52	13.530	8.06
Hexadecanoic acid, ethyl ester	14.372	5.97	14.356	1.80
Ethyl oleate	16.009	4.67	15.994	0.94
Octadecanoic acid, ethyl ester	16.231	1.36	16.224	0.24
Gamma-sitosterol	26.912	3.45	26.856	4.81
Gamma-sitostenone	29.296	2.33	29.243	3.87
Stigmastane-3,6-dione	33.141	0.90	33.138	1.13
Cyclopentane, 1-(3-methylbutyl)	9.226	0.91	9.222	1.30
Aromadendrene	10.056	1.64	10.050	2.88
Naphthalene,1,2,3,5,6,7,8,8A-octahydro-1,8A-dimethyl-7-(1-methylethenyl)	8.970	0.54	8.968	1.69
2,11-dioxatetracyclo[4,3,1,1(3,10,)0,(6,9)]undec-4-ene,3,7,7,10-tetramethyl	11.479	0.91	11.464	1.02
Isovalencenal	12.191	0.88	12.176	1.15
Sinularene	12.613	0.88	12.606	1.02
Stigmasta-3,5-diene-7-one	28.502	0.45	28.469	0.78
Cyclopentaneacetaldehyde,2-formyl-3-methyl-alpha-methylene	6.433	1.18	6.438	1.2
Actinidine	6.876	7.15	6.868	18.97
6-isopropenyl-4,8A-dimethyl-3,5,6,8,8A-hexahydro-1H-naphthalene-2-one	12.095	1.76	11.089	2.08
Coumarin-6-ol,3,4-dihydro-4,4,5,7-tetramethyl-methylsulfate(ester)	12.443	1.21	12.419	0.93
Linoleic acid ethyl ester (Ethyl linoleate)	15.948	2.40	15.937	0.49
Docosanoicacid,ethyl ester	19.536	3.08	19.529	0.18
Cyclopentaene,1-octyl	--	--	9.222	1.30
Megastigma-4,6(Z),8(E)-triene	--	--	12.081	1.55
3-Trifluromethylbenzoic acid, tetradecyl ester	--	--	13.286	1.81
Rescinnamine	--	--	20.686	4.91
Ergost-5-en-3-ol	25.559	0.29	25.546	0.55

Phytochemical analysis showed the presence of quite a lot of phytoconstituents including sesquiterpenes, sesquiterpenoids, esters, and steroids. The outcomes of the analysis confirmed that most of the phytoconstituents present in MCNJ were also reported in OMNJ.

The concentration of pharmacologically important sesquiterpenes such as spirojatamol (4.29%), jatamansone (7.86%), valeric acid (8.06%), valerenal (3.02%), globulol (6.71%), and steroidal compounds like sitosterol (4.81%), sitostenone (3.87%), stigmastane-3,6-dione (1.13%) stigmasta-3,5-diene-7-one (0.78%), and ergost-5-en-3-ol (0.55%) was found significantly higher in the extract obtained by using green approach (OMNJ). Also the concentration of cyclopentanoid monoterpene alkaloid, actinidine increased to more than twice in the OMNJ extract (~18%) as compared to the conventional extract, MCNJ (~7%). The possible justification for the increase in the quantity of these plant actives could be attributed to rupturing of the cell wall of the plant particles by microwave radiation. Further, it has also been reported that phytoconstituents like sesquiterpenes, steroids, ubiquinone are generally biosynthesized in the cytoplasm (Bick and Lange, 2003). Therefore disruption of the cell wall by microwave radiation causes the enhanced outward movement of accumulated secondary metabolite in the cytoplasm. Moreover, the monotepenes are generally presented in epidermal cells or trichome (Kutchan, 2005) so the significant increase in the concentration of actinidine by MAE is also justified. Because the microwave radiations vaporize the moisture present in the cell and resulted in rupturing of the cell membrane (Rostagno et al., 2009).

### 3.5 *In-Vivo* estimation of anti-alzheimer’s potential

*Nardostachys jatamansi* is a highly significant medicinal plant genera bearing wonderful memory enhancer, stimulant, and mind rejuvenator properties and is used in preventing various cognitive deficits and neurological disorders like Alzheimer’s disease (AD) (Bose et al., 2019). The majority of secondary metabolites present in the root extract of NJ are identified as antioxidants in nature and found to be tremendously effective in cognition enhancement (Saroya and Singh, 2018). Hence the optimized extract, OMNJ, along with the conventional extract, MCNJ, is also evaluated for anti-Alzheimer’s potential.

#### 3.5.1 Estimation of behavioral parameters

**Figure 9:**
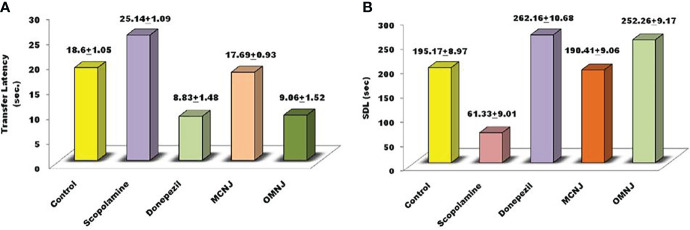
Effect of NJ extracts (MCNJ and OMNJ) on behavioral parameters **(A)** Transfer latency (TL) and **(B)**. Step down latency (SDL).

The parameters such as cognitive impairment, retention in learning, and memory power were analyzed using an elevated plus maze (EPM) and passive avoidance apparatus. Short-term memory impairment was observed by scopolamine administration in experimental animals. On the 15^th^ day, the transfer latency (TL) was noted for 5 min after drug administration for 24 h. The different extracts of NJ showed reduced effects on the TL in different groups for which the results are represented in **Figure 9A**. It was observed that TL for the scopolamine group was significantly increased in comparison to the control group (p<0.005). On treatment with extracts, the MCNJ and OMNJ reduced the TL significantly (p<0.05 and p<0.01 respectively) as compared to the negative control group. Also, the step-down latency (SDL) was found significantly decreased in the scopolamine-induced group as compared to the control group (p<0.005). **Figure 9B** indicated that SDL significantly improved by MCNJ (p<0.01) and OMNJ (p<0.01).

#### 3.5.2 Biochemical estimation of antioxidant parameters

**Figure 10:**
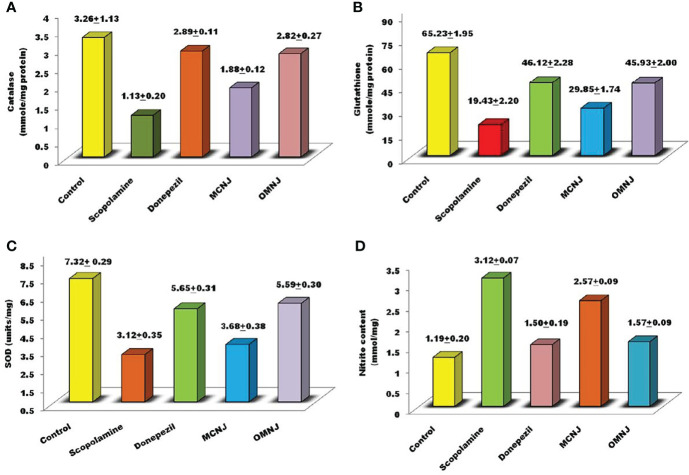
Biochemical evaluation of various extracts related to anti-oxidant stress **(A)** catalase **(B)** reduced glutathione **(C)** SOD **(D)** nitrite content.

Several studies have shown the involvement of oxidative stress in various pathogenesis mechanisms related to neurodegenerative disorders like AD. Also, the therapeutic strategies are based on preventing or reducing oxidative damage. Hence, in the present research, the effects of maceration and optimized MAE extracts on different biochemical parameters such as catalase and glutathione activity, nitric oxide content, and the superoxide dismutase concentration were also evaluated and the results were represented in **Figure 10**. Scopolamine significantly enhanced the oxidative stress level and also altered the concentration of glutathione, catalase, SOD, and nitric oxide content significantly as compared to the control group (p<0.005). The concentration of catalase, nitrite content (p<0.01), glutathione, and SOD (p<0.05) was significantly altered by OMNJ as compared to the negative control group. Conventional extract, MCNJ also reduced the catalase, SOD, and glutathione concentration but the change was not significant in the case of SOD. Various secondary metabolites including the phenolic and flavonoid compounds having antioxidant properties have been recognized as a part of therapeutic approaches beneficial in the management of AD (Devore et al., 2010; Feng and Wang, 2012). Antioxidants are linked with the decreased incidence of AD and a slower rate of cognitive impairment occurrence has been reported in patients of AD who are on high doses of antioxidants (93). The majority of phytoconstituents reported in roots of NJ comprises of sesquiterpenes like spirojatamaol, jatamansone, valeric acid, and globulol and steroidal compounds, and these compounds have been reported to exhibit anti-oxidant potential (Rice-Evans et al., 1995; Gilgun-Sherki et al., 2003; Melo et al., 2003; Bouayed, 2010; Neganova et al., 2012; Sharififar et al., 2012; Sharma and Singh, 2012; Hasnat et al., 2013; Razack et al., 2015; Goula et al., 2016; Saroya and Singh, 2018). The improved concentration of these metabolites could be responsible for the better anti-oxidant potential of OMNJ. Also, previous studies indicated that the scavenging of free radicals in the brain improves cognitive activities, prevents damage caused by oxidative stress, and shields CNS *via* BBB crossing (Bouayed, 2010). Thus, it can be interpreted from the data that this improvement anti-Alzheimer’s potential of the optimized extract could be a consequence of improved concentrations of various plant metabolites together with the TPC and TFC in OMNJ.

#### 3.5.3 AChE inhibitory potential of different extracts

**Figure 11:**
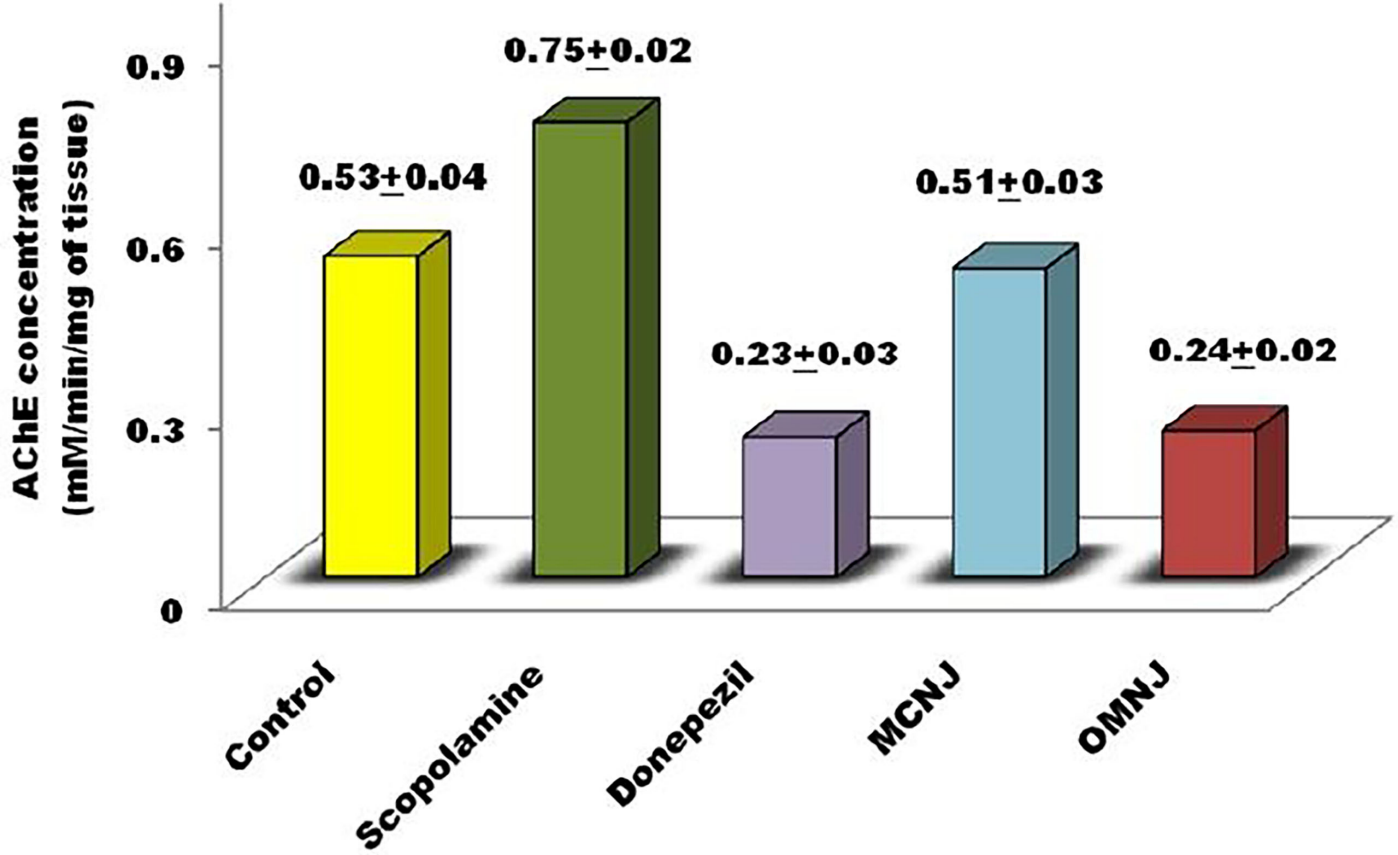
AChE inhibitory activity exerted by various extracts and standard drugs in different groups.

Acetylcholine neurotransmitter reduction was found to be associated with the degradation of cholinergic neurons which are present in the basal forebrain (Selkoe et al., 1994; Hamlin et al., 2013). The reduction of acetylcholine level leads to dementia which is found in patients who are critically suffering from AD (Nagele et al., 2002; Arya et al., 2021). Recent advancements in research emphasize the alteration of acetylcholinesterase (AChE) activity for AD management. As per the literature, AChE inhibitors reduce the symptoms associated with dementia by enhancing the acetylcholine release at cholinergic synapses, and also stimulate cognitive performance in humans and animals (Davidsson et al., 2001; Nagele et al., 2002; Devore et al., 2010; Rockwood, 2011). The different extracts were administered for fourteen succeeding days and it was observed that AChE activity significantly declined in all the extracts (**Figure 11**) when compared to the control (p<0.05 for MCNJ and p<0.01 for OMNJ).

With the help of data obtained from the experiments, we can easily predict that the optimized extract prepared by green methods (OMNJ) firmly possesses a higher potential of AChE inhibition in comparison to extracts obtained from conventional method owing to the presence of an improved concentration of secondary metabolites, mainly sesquiterpenes including TPC and TFC. These results are also concomitant with past studies which stated that various sesquiterpenes including that jatamansone inhibits AChE and can be used for the management of AD (Arya et al., 2021).

## 4 Conclusion

Worldwide an extreme inclination towards the use of herbal formulations has been observed over the last few decades. The *Nardostachys jatamansi* (D. Don) DC extracts or its polyherbal preparations hold a great demand in the global market. Its root extract has been used in several ayurvedic formulations like *Mentat* and *Intellimax* for the improvement of learning and memory power. Previously, the conventional methods were used to extract the herbs but now due to various limitations associated with these methods, the non-conventional and green techniques have been put forward to carry out the extraction of selected herbs. In the present study, roots of the NJ are extracted by a green method, MAE, at optimized conditions. MAE not only improves the kinetics of extraction but also enhanced the pharmacologically important sesquiterpenes (jatamansone, spirojatamol, globulol, valeric acid, etc) concentration as compared to conventional methods. Hence, it can be concluded that MAE could be employed for the extraction of roots of *Nardostachys jatamansi* at commercial scales after suitable modifications to meet the rising industrial demand.

## Data availability statement

The datasets presented in this study can be found in online repositories. The names of the repository/repositories and accession number(s) can be found below: Nil.

## Ethics statement

The animal study was reviewed and approved by Institutional Animal Ethical Committee, IAEC, Maharshi Dayanand University, Rohtak granted approval for our research protocol presented against vide reference number 1767/RE/S/14/CPCSEA/CAH/76-85 dated 26.02.2021.

## Author contributions

AA- Writing the original manuscript and conduct experiments. RC- Manuscript writing and conduct experiments. MA- Revise the manuscript and analyze the data. DK- Revise the manuscript and application of design software. LA- Manuscript revision and analyse the data. MK- Manuscript revision and data analysis. MA-D- Conceptualization, revision and analysis of data. VM- Conceptualization, Supervision, analysis and revision of manuscript.

## Acknowledgments

The authors want to sincerely acknowledge Maharshi Dayanand University, Rohtak, Haryana, India, and the Researchers Supporting Project number (RSP-2021/191), King Saud University, Riyadh, Saudi Arabia for their support.

## Conflict of interest

The authors declare that the research was conducted in the absence of any commercial or financial relationships that could be construed as a potential conflict of interest.

## Publisher's note

All claims expressed in this article are solely those of the authors and do not necessarily represent those of their affiliated organizations, or those of the publisher, the editors and the reviewers. Any product that may be evaluated in this article, or claim that may be made by its manufacturer, is not guaranteed or endorsed by the publisher.
